# The Association of *CD81* Polymorphisms with Alloimmunization in Sickle Cell Disease

**DOI:** 10.1155/2013/937846

**Published:** 2013-05-22

**Authors:** Zohreh Tatari-Calderone, Ryad Tamouza, Gama P. Le Bouder, Ramita Dewan, Naomi L. C. Luban, Jacqueline Lasserre, Jacqueline Maury, François Lionnet, Rajagopal Krishnamoorthy, Robert Girot, Stanislav Vukmanovic

**Affiliations:** ^1^Sheikh Zayed Institute for Pediatric Surgical Innovation, Children's National Medical Center, 111 Michigan Avenue NW, Washington, DC 20010-2970, USA; ^2^Department of Pediatrics, George Washington University School of Medicine and Health Sciences, Washington, DC 20052, USA; ^3^Immunology and Histocompatibility Department and INSERM, UMRS 940, Saint Louis Hospital, 75010 Paris, France; ^4^Divisions of Hematology and Laboratory Medicine, Children's National Medical Center, Washington, DC 20010, USA; ^5^Etablissement Français du Sang (EFS), Tenon Hospital, 75020 Paris, France; ^6^Centre de la Drépanocytose, Department of Internal Medicine, Tenon Hospital, 75020 Paris, France; ^7^INSERM, U763, Robert Debré Hospital, 75019 Paris, France; ^8^Hematology Department, Tenon Hospital, 75020 Paris, France

## Abstract

The goal of the present work was to identify the candidate genetic markers predictive of alloimmunization in sickle cell disease (SCD). Red blood cell (RBC) transfusion is indicated for acute treatment, prevention, and abrogation of some complications of SCD. A well-known consequence of multiple RBC transfusions is alloimmunization. Given that a subset of SCD patients develop multiple RBC allo-/autoantibodies, while others do not in a similar multiple transfusional setting, we investigated a possible genetic basis for alloimmunization. Biomarker(s) which predicts (predict) susceptibility to alloimmunization could identify patients at risk before the onset of a transfusion program and thus may have important implications for clinical management. In addition, such markers could shed light on the mechanism(s) underlying alloimmunization. We genotyped 27 single nucleotide polymorphisms (SNPs) in the *CD81*, *CHRNA10,* and *ARHG* genes in two groups of SCD patients. One group (35) of patients developed alloantibodies, and another (40) had no alloantibodies despite having received multiple transfusions. Two SNPs in the *CD81* gene, that encodes molecule involved in the signal modulation of B lymphocytes, show a strong association with alloimmunization. If confirmed in prospective studies with larger cohorts, the two SNPs identified in this retrospective study could serve as predictive biomarkers for alloimmunization.

## 1. Introduction

Transfusion of red blood cells (RBCs) is a key component of the comprehensive management of patients with SCD [1]. Transfusion increases the oxygen carrying capacity of blood by increasing hemoglobin A [2, 3] and decreasing hemoglobin S [4–7]. Further, transfusion decreases blood viscosity, improves blood flow, and suppresses endogenous erythropoiesis. Due to these benefits, most SCD patients receive multiple RBC transfusions in their lifetime. RBC transfusion therapy is complicated by development of antibodies specific for allelic (alloantibodies) or self (autoantibodies) RBC determinants. Alloantibodies are more frequent than autoantibodies, whose clinical significance remains questionable. The presence of anti-RBC antibodies in SCD patients may cause delay in finding suitable blood donors, which can result in life threatening anemia. In addition, anti-RBC antibodies may cause delayed hemolytic transfusion reactions, which resemble sickle cell crises and can be lethal [8–10]. Finally, anti-HLA antibodies promoting rejection of hematopoietic cell grafts are more frequent in patients with anti-RBC antibodies [11]. Identification of biomarker(s) predictive of susceptibility to alloimmunization could identify a subpopulation of SCD patients at risk before the onset of a transfusion program and thus has important implications for their clinical management. In addition, such markers could assist in enhancing our insight into the mechanism(s) underlying alloimmunization.

RBC alloantibodies develop in 18–47% of patients with SCD [3, 12–15]. These rates are higher than 0.2–2.8% found in transfused patients without SCD [16–19]. One possible explanation for these high rates is antigenic disparity-different blood group distribution between predominantly Caucasians blood donors and SCD patients who are of African or African-Caribbean descent [20]. This concept is supported by alloimmunization frequencies in SCD patients in Saudi Arabia (13.7%), Uganda (6.1%), Egypt (21.4%), and Tunisia (16.6%) where blood donors and SCD patients are from similar ethnic background [21–24]. Even lower alloimmunization rates (2.6%) were noted in a Jamaican patient cohort [25], but they might have been second to very low number of units received (1-2 per patient). However, even these reduced rates are higher than the above mentioned “background” rate of 0.2–2.8%, suggesting a multifaceted etiology of alloimmunization.

 The human population in Africa has been subject to selection pressures imposed by infectious agents. One of the best-documented examples is malaria which significantly shaped the genetic make-up in several regions of Africa [26]. The most prominent genetic trait is *Hbβ* ^*S*^ heterozygosity that confers about 10-fold increase in protection against life-threatening forms of plasmodium falciparum malaria [27]. Malaria infection has also exerted pressure for stronger immune responsiveness as evidenced by association of polymorphic variants in *HLA-B, HLA-DR, IL-4, CD40L, FcGR2A, and TNF*α** which result in increased resistance to malaria [26]. We reasoned that there may be other as yet undiscovered malaria-selected polymorphisms that promote stronger immune responsiveness and that some of them may lie close to *Hb*β**. Such neighboring immune response-modifying genetic markers are more likely to segregate with *Hbβ* ^*S*^ than those located farther away on the same chromosome, or those located on other chromosomes. We therefore hypothesized that two malaria-protective polymorphisms were coselected: the *Hbβ* ^*S*^ and an allele of a near-by gene, encoding a molecule with immunomodulatory function. The consequence of such coselection by heterozygosity would result in an undesired, exacerbated immune response in homozygous *Hbβ* ^*S*^ individuals, with an eventual increase in the incidence of alloantibody response following transfusion. The closest gene of immune interest to the *HBB *locus is the gene encoding Sjogren Syndrome Antigen 1. Although we have recently demonstrated an association of a SNP in this gene with tolerance induction in early childhood [28], we found no evidence of association with alloimmunization *per se,* which prompted us to continue our search for other genes in the neighborhood of Hb*β* gene. We herein identified a strong association of alloimmunization with two single nucleotide polymorphisms (SNPs) in the CD81 gene encoding for a molecule involved in the signal modulation of B lymphocytes which qualify them as predictive markers of alloimmunization.

## 2. Materials and Methods

### 2.1. Subjects

 Seventy-five adult SCD patients (44 Females, median age 32 years, range 21–73; 31 males, median age 30 years, range 20–58) regularly followed at Tenon Hospital in Paris, France were recruited for the study. All specimens were belonging to the larger French repository of blood samples from recipients of chronic RBC transfusion, financially supported and initiated in 1987 by the Institut National de la Transfusion Sanguine. Patients are first or second generation immigrants from Sub-Saharan Africa, that is, Atlantic West Africa, Central West Africa, and Bantu-speaking Africa (62 patients), and 13 patients were from the French West Indies. Patients were unrelated to each other and were not part of the same family. Samples were obtained during a routine clinical consultation at the steady state; a clinical status characterized by the absence of any infectious process or acute complication (such as vasoocclusive crisis) in the 3 months preceding the consultation. Patients were enrolled in this observational cross-sectional study after obtaining their informed consent according to the ethical research committee of “Assitance Publique-Hopitaux de Paris (AP-HP)” at Tenon Hospital. The criteria for inclusion were: (1) the diagnosis of sickle cell anemia (made using standard laboratory procedures including complete blood count, hemoglobin electrophoresis and family studies, and by direct molecular identification of the Hb*β* mutation) and (2) having received at least five RBC transfusions matched for AB0 and RhD blood antigens. Alloimmunized patients were received antigen negative blood for identified alloantibodies [40]. All patients received RBC transfusions during their in- or outpatient treatment at Tenon Hospital. Leukoreduced RBC units were collected and distributed by “Etablissement Francais du Sang (EFS).” Patients receiving hydroxyurea therapy (HU) were identified in each group to evaluate the influence of this therapy on the incidence of alloimmunization (Table 1). The mean ages of the patients at the time of the study were 35.1 ± 8.4 years (mean ± SD) and 30.9 ± 8.7 years (mean ± SD) for patients with and without alloimmunization, respectively (Table 1). The mean of age, gender, number of RBC transfusions and exposure to hydroxyurea did not vary significantly between alloimmunized and nonalloimmunized patients.

### 2.2. SNP Detection

 Genotyping was performed using a TaqMan allelic discrimination assay that employs the 5′ nuclease activity of Taq polymerase to detect a fluorescent reporter signal. Allele-specific oligonucleotides designed by Applied Biosystems, (Foster City, CA, USA) were labeled with different fluorophores in order to detect both alleles simultaneously. Genotypes were determined by the ratio of the two fluorophores used. The PCR for each SNP contained 20 ng of DNA, 900 nM primers, 200 nM probes, and TaqMan Universal PCR Master Mix, in a final volume of 15 *μ*L. PCR was performed on an MJ Research Tetrad thermal cycler (Waltham, MA, USA). The PCR profile was 10 minutes at 95°C (denaturation), 44 cycles of 15 seconds at 92°C, and 1 minute at an annealing temperature of 60°C. Reactions were set up using a MWG robot, and fluorescence ratios and allele calling were done using an ABI 7900. For the quality control of our genotyping, 10% of samples were randomly tested in three independent setup, and genotypes were compared. There was 100% concordance of genotypes in all three replicates.

### 2.3. *β*-Globin Gene Locus Genotype

 Beta-globin gene haplotypes were determined by PCR-RFLP as previously described [43].

### 2.4. Statistical Analysis

The association of SNPs with alloimmunization was performed using the Chi-square testing with Yate's correction or Fisher's exact test when appropriate. After Bonferroni adjustment for multiple testing, the findings were considered statistically significant if *P* value was equal or less than 0.00185. 

## 3. Results

### 3.1. Alloantibodies

 Thirty-five of 75 adult patients (47%) had clinically significant RBC alloantibodies, listed in Table 2 (except for the Le^a^ and Le^b^-specific antibodies that were considered clinically not significant). The incidence of alloimmunization in males (14 of 31 males who received transfusion were 45%) and females (21 of 44 females who received transfusion were 48%) was not significantly different. The number of alloantibodies per patient ranged from 1 to 9. Twenty patients had one alloantibody, eight patients had two, and nine patients had three or more antibodies. In twenty-eight patients (80%) who developed significant alloantibodies, the specificity was directed against antigens in the Rh or Kell systems. Five patients developed antibodies in the Rh system with uncommon specificity (C^w^, partial D, partial C, V, and rh_i_). However, twenty one patients (60%) developed additional alloantibodies to antigens in the Duffy, Kidd, Lewis, and MNS systems, and six patients (17%) developed additional antibodies to antigens in uncommon blood group systems.

### 3.2. Selection of SNPs for Analysis

There are several genes located in the vicinity of the *HBB *locus that can potentially impact the immune system (Table 3), with literally hundreds of SNPs identified in each. Given the plethora of candidate SNPs, we identified two sets of criteria to establish the priority for SNP analysis as following.

1. Criteria related to the selection of genes:
  - closeness of the gene to the *Hbβ* globin gene (the closer the genes are, the more likely the alleles are in linkage disequilibrium (LD));
  - involvement of the gene product in immune response;
  - preserved homology of the genes between species (the less homologous genes are likely to produce a “high background” of genetic variation against which it is more difficult to detect genetic associations).
2. Criteria related to the SNPs selection:
  - known association of a polymorphism with an immune phenomenon;
  - SNP frequency difference (HapMap database) between Yoruba (YRI) in Ibadan, Nigeria, and CEPH (Utah residents with ancestry from northern and western Europe: CEU) [44];
  - characterization of SNPs by the HapMap database as informative for the linkage disequilibrium in haplotype analysis using the software Haploview version 3.2 (http://www.broadinstitute.org/). The HapMap database provides information on millions of SNPs that determine the variation among human beings. This database provides the frequency for each SNP located in the noncoding region of the genome.

Based on the above criteria, we examined HapMap-designated-informative SNPs in *ARHG*, *CHRNA10,* and *CD81* genes. 

### 3.3. Association of CD81 SNPs and Alloimmunization

Table 4 summarizes the results of association analysis of alloimmunization with 27 SNPs, all located in the noncoding regions of appropriate genes. Overall, allelic distribution was similar to that reported in the general population in Sub-Saharan region (HapMap; NCBI). For some patients, we were unable to establish a genotype despite repeated attempts. These patients may have previously undescribed/novel alleles or additional polymorphisms in the primer-binding regions. Therefore, the total number of patients for some SNPs do not equal the 35 (alloimmunized) or 40 (nonalloimmunized). Of the 27 SNPs studied, two had a statistically significant association with alloimmunization. Both SNPs are located in the CD81 gene and, based on the haploview analysis of disequilibrium linkage data, are in general not coinherited. Consistent with this, the “informative” genotypes (defined as those that differ most between the alloimmunized and nonimmunized patients-CC in rs708564 and CC in rs2237863) were not coinherited in most patients. Of the 28 patients with rs708564 C/C genotype, 9 also had the rs2237863 C/C genotype. The presence of at least one of the “informative” genotypes was present in 37 out of 75 patients (49%). The presence of “informative” genotypes correctly “predicted” alloimmunization status in 30 out of 40 SCD patients (75%) with no antibodies detected, and only in 6 of 35 patients (17%) with antibodies (Table 5). The absence of both two “informative” genotypes occurred in 29 out of 35 patients with antibodies (83%) and in 10 out of 40 patients without antibodies (25%). Thus, these SNPs may serve as a powerful predictor of alloimmunization.

### 3.4. Hemoglobin *β* Haplotypes

 The existence of five *Hbβ* ^*S*^ haplotypes suggested that the *Hbβ* ^*S*^ mutation arose independently in different geographical regions [45, 46]. The coinheritance of *Hbβ* ^*S*^ neighboring DNA markers, including the ones affecting alloimmunization, could also have selectively occurred in one or several independently derived haplotypes. Therefore, we studied the *Hbβ* ^*S*^ haplotypes in this patient cohort. Only patients homozygous for Bantu and Benin haplotypes were present in numbers sufficient to allow statistical analysis (Table 5). The predictive power of rs708564C/C and/or rs2237863C/C CD81 SNP genotypes was preserved in subgroups of patients with Bantu/Bantu, Benin/Benin, or all other *Hbβ* ^*S*^ haplotypes grouped together. These data suggest that CD81 SNPs in our subject population were inherited independently of any particular *Hbβ* ^*S*^ haplotype.

## 4. Discussion

Blood and blood product transfusion most frequently either provoke no obvious immune reaction or induce immune suppression. The latter, known as transfusion-related immunomodulation, may reduce transplant rejection rate and promote cancer recurrence, postoperative infections, and virus activation [47, 48]. So, what is (are) the factor(s) in some transfusion settings that converts an inert or tolerogenic event into an immunogenic one, leading to alloimmunization? And why the alloimmunization rates are higher in subjects with SCD? One theory is that discrepancies in RBC antigen protein structures between blood donors and recipients enhance the stimulation of B-cell clones to produce glycoprotein-specific alloantibodies. Blood group distribution is different between predominantly Caucasians blood donors and SCD patients who are of African or African-Caribbean descent. The SCD patients in the present study are mostly first/second generation Africans or West Indies whereas blood donors are mostly Caucasians. Hence, the antigen disparity between donor and recipients can explain at least in part the relatively high rate of alloimmunization (44%) in our patient population. 

Transfusion-nonrelated preexisting recipient conditions that may lead to increased inflammation and activation of innate immunity are thought to contribute to alloantibody development [49, 50]. This has certainly been shown in a mouse model of alloimmunization [51]. SCD patients display increased inflammation and activation of innate immunity [52, 53] and increased levels of serum cytokines [54–57]. While antigenic disparity and proinflammatory milieu are important factors for higher rates of alloimmunization in SCD, it still remains unclear why the majority of RBC recipients never develop allo-/autoantibodies despite multiple transfusions. We therefore hypothesized that immune response to RBC alloantigens is additionally influenced by inheritable variations of immune function. We searched for genetic variations in the vicinity of Hb*β* locus because one of the several susceptibility loci for development of autoimmune/inflammatory diseases is located on chromosome 11p15, in proximity of Hb*β* [58]. Although the present work does not constitute a proof for the above hypothesis, it provides the first of several steps towards that goal. If confirmed in larger patient cohorts, the work may lead to the discovery of markers of alloimmunization-genetic traits that associate with alloimmunization, but do not necessarily cause or prevent alloimmunization. Although *CD81* is in the relative vicinity of the Hb*β* locus, the distance is large enough to suggest that the linkage disequilibrium is not a likely mechanism of association.

CD81 is a molecule which participates in the formation of so-called tetraspanin web that promotes cisassociations with different cell surface receptors including CD19/CD21 in B cells CD4 and CD8 in T cells [59, 60]. This association has functional consequences in B-cell-related pathways. Indeed, enhanced activation is observed in CD81 deficient B cells [32] and the use of antibodies to CD81 alter CD19/CD21-mediated signaling [33]. In addition, CD81 is a part of a complex involved in the uptake of exosomes by dendritic cells [61] and can modulate chemokine-induced dendritic cell migration [62]. Thus, CD81 can influence the immune system at multiple levels,b c and the identified SNPs may affect the function of CD81 protein at any of these steps. An alternative explanation is that markers may have an indirect effect on CD81, or may be associated with alloimmunization through linkage disequilibrium to a yet to be identified functional variant within CD81 gene or another gene.

SCD is caused by homozygous mutation in a single gene (Hb*β*, glu6val), but it has exceptional phenotypic variability. This is explained by the interaction of environmental factors with a select group of polymorphic genes. Understanding whether there are genetic modifiers responsible for the phenotypic variability in clinical disease manifestations may provide novel approaches to treatment. The most studied genetic modifiers affecting the severity of SCD are the level of *HbF* expression, BCL11A, HBS1L-MYB, and coinheritance of **α*-thalassemia* [63–65]. Other modifier genes include *IL-4R*, *TGF*β*R3*, adenylate cyclase, and HLA-DPB1*0401 where certain allelic variants predispose SCD patients to large vessel stroke [26, 66, 67]. SNPs in bone *morphogenetic protein receptor 2 (BMPR2)* is associated with familial and sporadic pulmonary hypertension [68–70], and acute chest syndrome in women with SCD is associated with a SNP in the endothelial *nitric oxide synthase* gene (*NOS3*) [71]. Sickle cell adhesion to laminin may be partially dependent on SNPs in the **β*-adrenergic receptor gene (ADRB2)* and *adenylate cyclase (ADCY6)* gene [72]. Susceptibility to infection in SCD has been associated with polymorphism in HLA class II gene. The data shows a protective role of HLA-DRB1*15 against infection, in contrast to HLA-DQB1*03 which predisposes the SCD patients to a higher risk of infection such as meningitis, septicemia and osteomyelitis [73]. In contrast, some polymorphisms in *TNF*α**, **β*-adrenergic receptor 2 (ADRB2)* and HLA-DPB1*1701 were protective against stroke [74]. Given the plethora of studies addressing genetic influences on different aspects of SCD, it is surprising that genetic modulation of alloimmunization has been virtually unexplored. One study has reported the possible association between *HLA-B35* and RBC alloantibodies [75]. However, the study was performed on a very small number of subjects, and the finding has not been replicated. 

## 5. Conclusions

Our data suggest that SNPs in *CD81* gene could potentially serve as predictors of RBC alloimmunization. The present study will have to be expanded in independent cohort of patients such as African-American patients with SCD. Furthermore, the impact of the SNPs on CD81 expression, and the role of CD81 in alloimmunization will need further investigation to gain full understanding of the mechanism underlying the cis-association of rs708564 and rs2237863 with alloimmunization.

## Figures and Tables

**Table 1:** Clinical and demographic characteristics of SCD patients.

	Alloimmunized	Non-alloimmunized	*P* value
	(*N* = 35)	(*N* = 40)	
Age			
Mean age ± SD	35.1 ± 14	30.9 ± 8.7	NS
Range	(21 y–73 y)	(20 y–58 y)	
Gender			
Male	14	17	NS
Female	21	23	
RBC transfusion			
<30 unit	25	25	NS
>30 unit	10	15	
Hydroxyurea			
Yes	11	17	NS
No	24	23	

**Table 2:** Distribution and specificities of alloantibodies.

Blood group system	Alloantibody specificity	Number of alloantibodies
Rh	D, D partial, C, C partial, c, C^w^, E, rh_i_, and V	24
Kell	K and Kp^a^	4
Duffy	Fy^a^, Fy^a,b^, and Fy5	9
Lewis	Le^a^ and Le^b^	1
Kidd	Jk^a^ and Jk^b^	7
MNS	M and S	4
Dombrock	H_y_	1
Colton	Co^b^	1
Cartwright	Yt^b^	1
Other blood group systems	Kn^a^, Kn^b^, and Bg^a^	3

**Table 3:** List of genes in the proximity of HBB locus encoding molecules affecting the function of the immune system.

Gene symbol	Distance from HBB locus (MB)	Molecule	Effects on the immune system	Sequence homology			
				*P. troglodytes *	*M. musculus *	*Rattus norvegicus *	*Canis familiaris *
*SSA1 *	0.83	SSA1, Ro52, and TRIM21	Encodes Ro52 antigen in Sjogren Syndrome and lupus [29]	99	69	68	67
*ARHG *	1.38	RhoG	Stronger immune function in knock-out mice [30]	100	100	100	99
*CHRNA10 *	1.55	*α*10 subunit of AchR	Modulates T-cell Ca^2+^ mobilization [31]	100	91	90	88, 92*
*CD81 *	2.85	CD81	Modulates BCR signaling [32, 33]	99	91	93	83
*PHEMX/TSSC6 *	2.92	TSSC6	Higher T-cell responses in knock-out mice [34]	NA	63	57	NA
*LSP1 *	3.41	LSP1	Regulation of B cell apoptosis [35]	100	67	64	82, 88*
*TOLLIP *	3.95	Toll inh. prot.	Downmodulates IL-1 and TNF*α* signalings [36]	72	93	93	67
*CD151 *	4.39	CD151	Represents blood group antigen MER-2 [37]	98	93	92	94
*IRF7 *	4.60	IRF7	Interferon production [38]	96	62	63	NA
*SIGIRR *	4.80	SIGIRR	Negative regulator of IL-1 signaling [39]	98	73	82	NA

*Only partial sequence of canine *CHRNA10* and *LSP1* genes is available. Distinct numbers represent homology of different available gene segments.

**Table 4:** SNP genotype association with alloimmunization in SCD.

SNP	Gene	Genotypes	Number of patients*		*P* value	HapMap^#^
			Antibody positive	Antibody negative		
(1) rs1049388	*ARHG *	CC/CG/GG	21/8/0	21/11/0	0.593273	62/30/8
(2) rs1451724	*ARHG *	AA/AG/GG	1/3/27	0/3/29	0.833054	0/23/77
(3) rs4910852	*ARHG *	AA/AG/GG	25/7/0	18/10/0	0.264449	61/30/9
(4) rs7128013	*ARHG *	AA/AC/CC	13/12/5	11/18/4	0.543399	25/49/26
(5) rs7929197	*ARHG *	CC/CT/TT	7/13/12	8/12/5	0.429608	30/45/25
(6) rs10742177	*ARHG *	CC/CG/GG	10/14/7	18/3/5	0.008979	41/45/14
(7) rs10835182	*ARHG *	AA/AT/TT	0/14/15	0/12/20	0.444898	2/23/75
(8) rs10835184	*ARHG *	AA/AT/TT	25/2/0	26/2/2	0.550071	95/5/0
(9) rs12272393	*ARHG *	AA/AC/CC	7/13/14	8/12/11	0.817062	30/45/25
(10) rs17173879	*ARHG *	AA/AG/GG	30/2/0	25/1/0	0.999999	93/7/0

(11) rs2231529	*CHRNA *	CC/CT	27/3/1	35/2/0	0.493847	93/7/0
(12) rs2231532	*CHRNA *	AA/AG/GG	19/13/3	14/12/5	0.609984	63/35/2
(13) rs2271583	*CHRNA *	AA/AG/GG	4/14/15	2/9/18	0.422893	10/33/57
(14) rs2672213	*CHRNA *	CC/CT/TT	30/2/0	23/4/0	0.397881	97/3/0
(15) rs2672216	*CHRNA *	CC/CT/TT	32/2/0	25/8/1	0.062890	85/13/2
(16) rs2741862	*CHRNA *	CC/CT/TT	5/25/4	14/14/5	0.022396	23/35/42
(17) rs12221525	*CHRNA *	CC/CG/GG	20/8/4	20/11/2	0.562563	50/37/13

(18) rs708564**	***CD81 ***	**CC/CT/TT**	**5/15/11**	**23/8/3**	**0.000086** ^ ##^	**82/17/2**
(19) rs731909	*CD81 *	CC/CG/GG	27/6/3	33/2/0	0.064002	90/8/2
(20) rs756915	*CD81 *	CC/CT/TT	9/19/8	12/14/10	0.495260	21/50/29
(21) rs800137	*CD81 *	CC/CT/TT	6/16/13	14/14/7	0.086688	46/31/24
(22) rs800335	*CD81 *	CC/CT/TT	7/10/18	4/8/24	0.374866	82/17/2
(23) rs874330	*CD81 *	CC/CT/TT	13/15/7	19/9/7	0.284287	52/40/8
(24) rs2019938	*CD81 *	AA/AG/GG	11/13/5	15/14/8	0.851861	43/33/23
**(25) rs2237863**	***CD81 ***	**CC/CT/TT**	**3/22/5**	**15/10/6**	**0.001518** ^ ##^	**15/47/38**
(26) rs11022565	*CD81 *	GG/GT/TT	17/14/4	21/7/5	0.280369	61/32/7
(27) rs11022567	*CD81 *	AA/AG/GG	5/14/16	5/7/25	0.120284	7/33/60

*Patient numbers are displayed in the order shown in the column labeled genotypes. ^#^Relative distribution of genotypes in Sub-Saharan population, as reported by the HapMap. This research utilizes the NCBI SNP database [41], (http://www.ncbi.nih.gov/snp/). The Single Nucleotide Polymorphism database (dbSNP) is a public domain archive for a broad collection of simple genetic polymorphisms. dbSNP reports many cases of SNPs genotyped by HapMap and other projects which provide additional genotype and allele frequency information. **NCBI database describes two additional alleles, A/G with very low frequencies (<0.5%) for rs708564. This might be due to a mutational mechanism that leads to the simultaneous creation of two new base pairs at the same site which is beyond the scope of this study [42]. Our results, however, are based on the two reference SNPs alleles (C/T) of rs708564. ^##^ *P* values significant comparing SCD patients with and without alloimmunization.

**Table 5:** Hb*β* ^*S*^ genotypes in SCD patients with or without antibodies or with “informative” SNP genotypes.

Hb*β* ^*S*^ haplotype	Total patients	Antibody positive		Antibody negative		*P* value
		At least one C/C genotype*	No C/C genotypes*	At least one C/C genotype*	No C/C genotypes*	
Bantu/Bantu	15	2	6	7	0	0.0069
Benin/Benin	28	1	10	12	5	0.0021
Other**	33	3	13	11	5	0.0113

Total	75	6	29	30	10	6.17 × 10^−7^

*Patients were divided into groups that had rs708564C/C and/or rs22378563C/C genotypes (group one) or were double negative for these genotypes (group two).

**Includes any haplotypes found in 10 or less subjects (Senegal/Senegal; Benin/Cameroon; Senegal/Benin; Cameroon/Cameroon; Benin/Bantu; Bantu/Cameroon; Senegal/Cameroon; Benin/*β*thal; and Senegal/*β*thal).

## Abbreviations

Hb: Hemoglobin
RBCs: Red blood cells
RFLP: Restriction fragment length polymorphism;
SCA: Sickle cell anemia
SCD: Sickle cell disease.
